# Validation of a novel particle isolation procedure using particle doped tissue samples

**DOI:** 10.1016/j.dib.2018.04.096

**Published:** 2018-05-01

**Authors:** J. Patel, S. Lal, S.P. Wilshaw, R.M. Hall, J.L. Tipper

**Affiliations:** a Faculty of Biological Sciences, University of Leeds, UK; b School of Mechanical Engineering, University of Leeds, UK

## Abstract

A novel particle isolation method for tissue samples was developed and tested using particle-doped peri-articular tissues from ovine cadavers. This enabled sensitivity of the isolation technique to be established by doping tissue samples of 0.25 g with very low particle volumes of 2.5 µm^3^ per sample. Image analysis was used to verify that the method caused no changes to particle size or morphologies.

**Specifications table**

**Table:** 

Subject area	Biology
More specific subject area	Biomaterials
Type of data	Figures, graphs, table
How data was acquired	Scanning electron microscopy and image analysis with imageJ software
Data format	Raw and in tabulated and histogram format
Experimental factors	Tissue samples from animal cadavers (ovine) were formalin fixed and stored in 70% (v/v) ethanol. The tissue samples were doped with 2.5 µm^3^ of silicon nitride particles, or 0.1 mm^3^ of CoCrMo or Ti-6Al-4V particles using a microbalance to weigh particles
Experimental features	Samples were subjected to the particle isolation process given in [1] and isolated particles were imaged and measured
Data source location	N/A
Data accessibility	Data is with this article
Related research article	[1]

**Value of the data**

• The data may be used to draw comparisons between the size and morphology of different particles.
• The data give an indication for the sensitivity (minimum amount of particles) that has been achieved using the particle isolation method presented in Ref. [1].
• The data provide evidence that the method presented in Ref. [1] does not have a measureable effect on the size or morphology of particles.

## 1 Data

The data presented here are the SEM micrographs of particles before and after isolation, elemental analysis of the particles, particle size distributions before and after isolation, and average particle parameters before and after isolation. Below is a description of the results obtained.

**Fig. 1:**
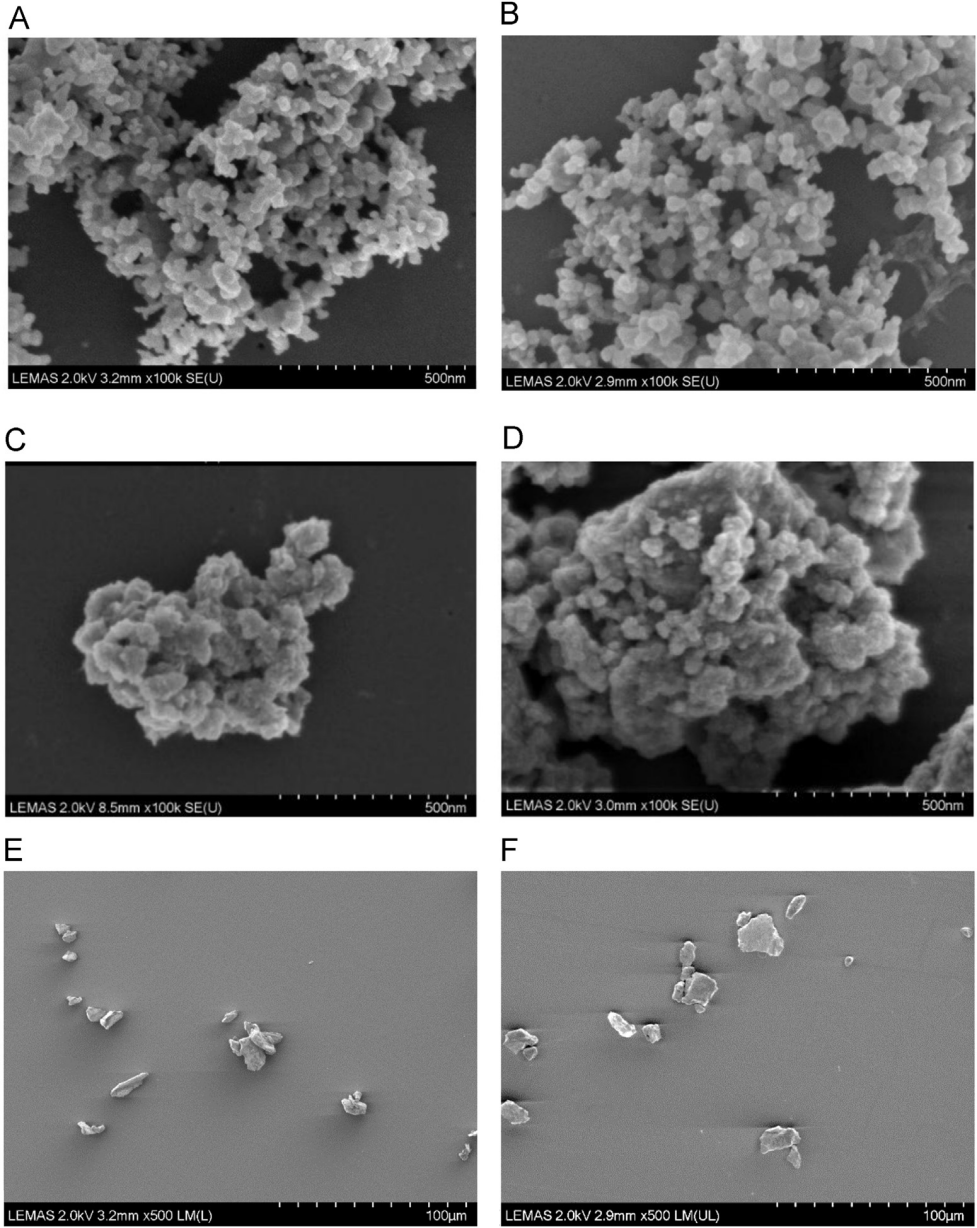
Aggregates of commercial Si_3_N_4_ particles (A and B), CoCrMo particles (C and D) or Ti-6Al-4V particles (E and F), not subject to isolation (left) and after isolation (right), imaged using CFE-SEM.

The Si_3_N_4_ particles were observed as aggregates of approximately 0.2–2 µm in size distributed across the filter membrane (Fig. 1A and B). The particles were relatively spherical and were nanoscale. The CoCrMo particles also formed small aggregates of approximately 0.2–2 µm in size distributed across the filter membrane, and particles were also relatively spherical and nanoscale (Fig. 1C and D). Titanium particles showed no aggregation characteristics, were micron scale and were less spherical in shape (Fig. 1E and F). For each material, the isolated particles were similar to the non-isolated particles, and isolated particles were observed to be free from protein contamination. Within the material groups, each replica sample of isolated particles was similar. Particles were absent from the control samples.

**Fig. 2:**
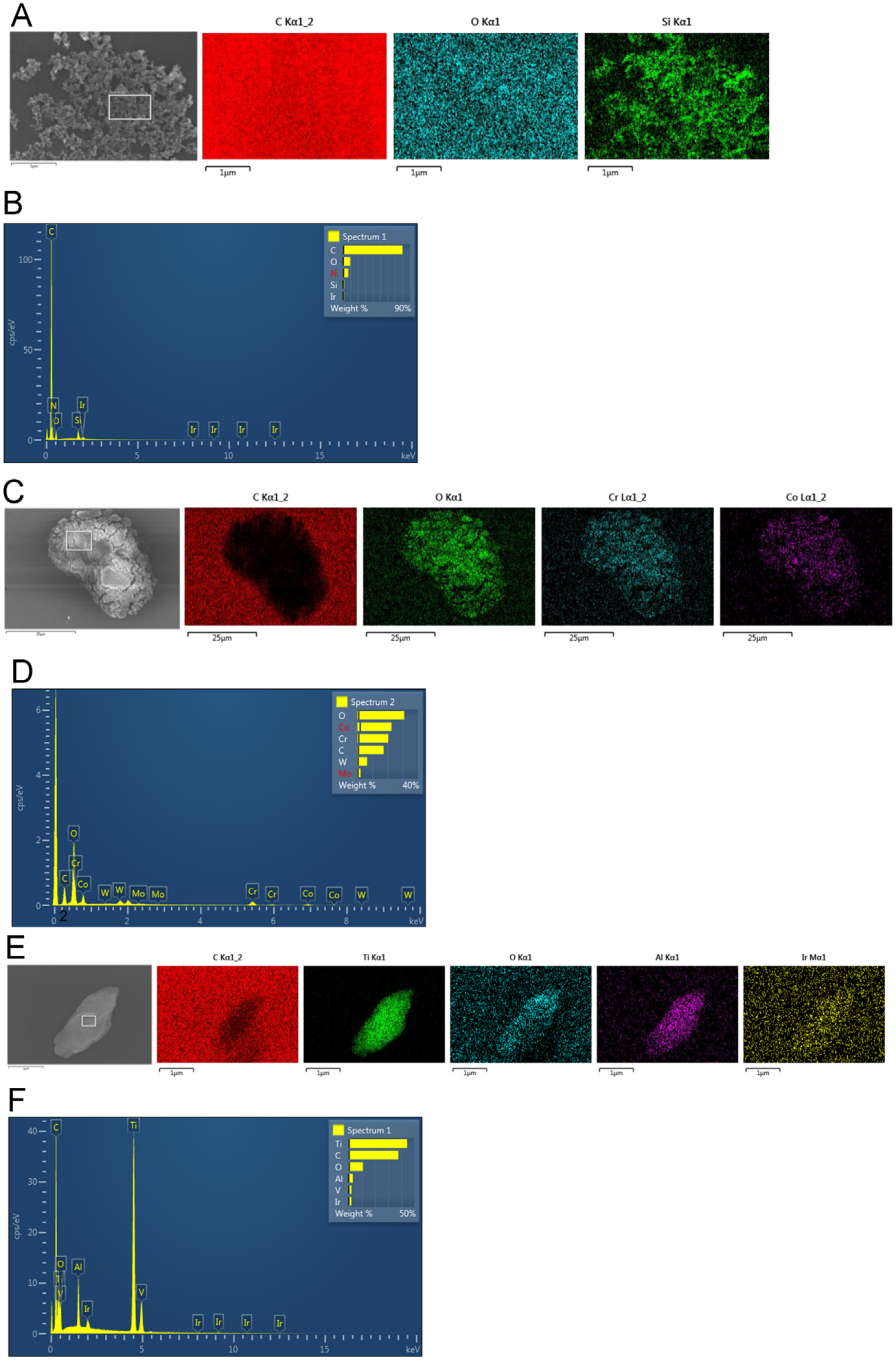
Elemental analysis of isolated Si_3_N_4_ particles (A and B), isolated CoCrMo particles (C and D) and isolated Ti-6Al-4V particles (E and F).

Elemental analysis was carried out on the isolated particles as shown by EDX maps and spectrums (Fig. 2). Oxygen, iridium and carbon signals were detected in the polycarbonate filter membrane and sputter coating. Elemental maps demonstrated that silicon was detected exclusively in areas containing silicon nitride particles (Fig. 2A). This was confirmed by spectrum analysis, which also demonstrated the presence of nitrogen within the particles (Fig. 2B). Elemental maps of cobalt chromium particles demonstrated that chromium and cobalt signals originated exclusively in areas containing particles (Fig. 2C). However, traces of tungsten were detected on the cobalt chromium particles using spectrum analysis (Fig. 2D). Oxygen was also detected within the particles, suggesting that particles were oxidized during filtration. This was not very apparent in the silicon nitride particles. Titanium particles were also verified by elemental maps (Fig. 2E), which identified the presence of titanium and aluminium exclusively in particles; spectrum analysis also demonstrated the presence of vanadium (Fig. 2F). However, as with the cobalt chromium particles, a degree of particle oxidation was apparent. Elemental analysis was similar in non-isolated particles; however, tungsten was not present on the pristine CoCrMo particles.

**Fig. 3:**
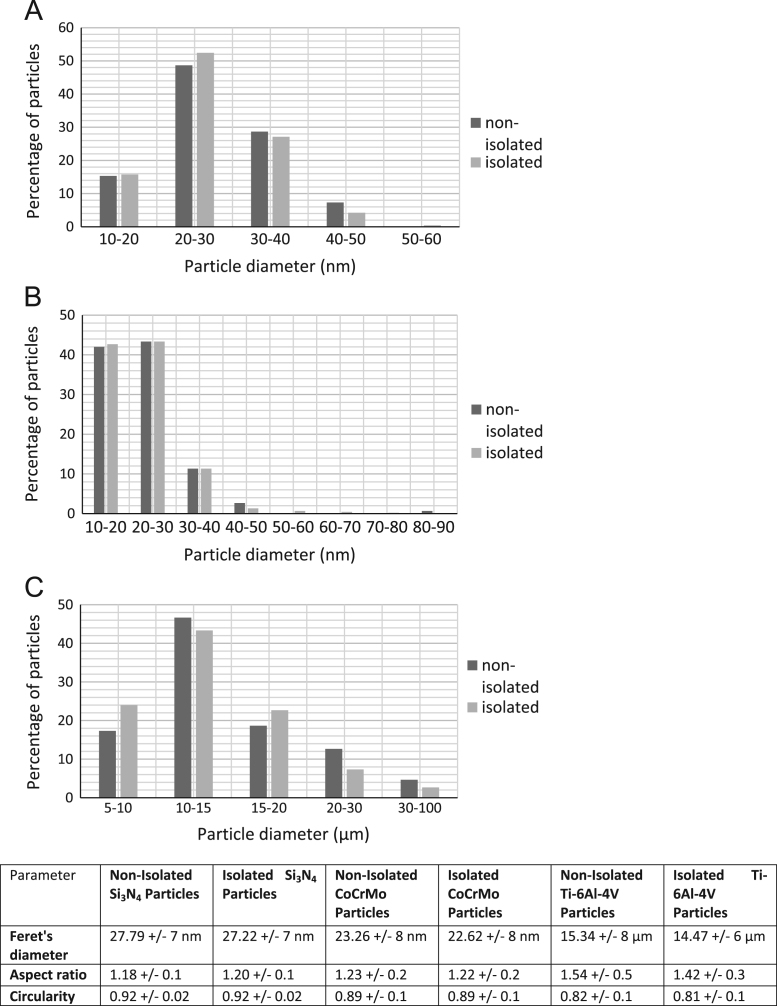
Particle size distributions for (A) Si_3_N_4_, (B) CoCrMo and (C) Ti-6Al-4V, and mean particle characteristics for both isolated and non-isolated particles, ±standard deviation.

Isolated and non-isolated particles had similar size distributions, average particle sizes, aspect ratios and circularities (Fig. 3). Particle characterisation showed that Si_3_N_4_ particles had a size range of 10–60 nm and a modal size of 20–30 nm (Fig. 3A). CoCrMo particles were 10–90 nm in size, and most were 10–30 nm (Fig. 3B). Titanium particles were 5–100 µm in size, with a modal particle size of 10–15 μm (Fig. 3C). The low aspect ratio and high circularity of both Si_3_N_4_ and CoCrMo particles indicated that particles were relatively spherical; titanium particles were less spherical (Fig. 3D). Statistical analysis showed no significant changes to any of the particle parameters for any of the material groups before and after isolation (KS tests, *p*>0.5).

## 2 Experimental design, materials, and methods

Ti-6Al-4V and CoCrMo particles were generated using pins and plates as described in [2]. Commercial Si_3_N_4_ particles (<50 nm, Sigma-Aldrich, UK) were also used.

Ovine capsular tissues consisting of fat, ligament and synovium were harvested from cadavers, formalin fixed, stored in 70% (v/v) ethanol, washed three times in sterile filtered water, immediately discarding each wash, and minced to 1 mm^3^. Three replica 0.25 g tissue samples were doped with a volume of 1 mL of a 2.5 µm^3^ mL^−1^ Si_3_N_4_ particle suspension. Particle suspensions were vortexed and sonicated for 20 min three times prior to use to ensure a homogenous dispersion. The tissue samples were incubated with particles on an orbital shaker overnight at 37 °C to simulate *in vivo* conditions and enable protein to bind to particles. The rest of the protocol was performed as detailed in Ref. [1]. The experiment was repeated in three replica 0.25 g tissue samples doped with a volume of 1 mL of a 25 µm^3^ mL^−1^ CoCrMo particle suspension, and in three replica 0.25 g tissue samples doped with a volume of 1 mL of a 25 µm^3^ mL^−1^ Ti-6Al-4V particle suspension. For each of the three material groups, a particle-free control sample of 0.25 g of tissue was included and processed in the same way. Particle characterisation was performed as detailed in Ref. [1]; however, in the titanium group, 50 isolated particles from each sample were measured to give a total of 150 particles. In Si_3_N_4_ and CoCrMo groups, 150 isolated particles from each sample were measured by ImageJ to give a total of 450 isolated particles per material group.

The data for each parameter (size, aspect ratio, circularity) from samples of non-isolated particles was compared statistically to the data from the isolated particles using Kolmogorov-Smirnov tests (IBM SPSS, v23). In each case the data from 150 non-isolated particles were used.
