# Supplementary material for: Validation of a novel particle isolation procedure using particle doped tissue samples
Source: Data Brief. 2018 May 1;18:1802–7. doi: 10.1016/j.dib.2018.04.096 (PMC5998301; doi:10.1016/j.dib.2018.04.096)
Supplement: Supplementary file 1 — Supplementary material [file mmc1.docx]

**Declaration of interest**

None.
